# Perceptions of Information and Communication Technology as Support for Family Members of Persons With Heart Failure: Qualitative Study

**DOI:** 10.2196/13521

**Published:** 2019-07-16

**Authors:** Hanna Allemann, Ingela Thylén, Susanna Ågren, Maria Liljeroos, Anna Strömberg

**Affiliations:** 1 Department of Medical and Health Sciences Linköping University Linköping Sweden; 2 Department of Cardiology Linköping University Linköping Sweden; 3 Department of Cardiothoracic Surgery Linköping University Linköping Sweden; 4 Centre for Clinical Research Sörmland Uppsala University Eskilstuna Sweden

**Keywords:** family, caregivers, telemedicine, perception, heart failure, social support, focus groups, qualitative research

## Abstract

**Background:**

Heart failure (HF) affects not only the person diagnosed with the syndrome but also family members, who often have the role of informal carers. The needs of these carers are not always met, and information and communications technology (ICT) could have the potential to support them in their everyday life. However, knowledge is lacking about how family members perceive ICT and see opportunities for this technology to support them.

**Objective:**

The aim of this study was to explore the perceptions of ICT solutions as supportive aids among family members of persons with HF.

**Methods:**

A qualitative design was applied. A total of 8 focus groups, comprising 23 family members of persons affected by HF, were conducted between March 2015 and January 2017. Participants were recruited from 1 hospital in Sweden. A purposeful sampling strategy was used to find family members of persons with symptomatic HF from diverse backgrounds. Data were analyzed using qualitative content analysis.

**Results:**

The analysis revealed 4 categories and 9 subcategories. The first category, about how ICT could provide relevant support, included descriptions of how ICT could be used for communication with health care personnel, for information and communication retrieval, plus opportunities to interact with persons in similar life situations and to share support with peers and extended family. The second category, about how ICT could provide access, entailed how ICT could offer solutions not bound by time or place and how it could be both timely and adaptable to different life situations. ICT could also provide an arena for family members to which they might not otherwise have had access. The third category concerned how ICT could be too impersonal and how it could entail limited personal interaction and individualization, which could lead to concerns about usability. It was emphasized that ICT could not replace physical meetings. The fourth category considered how ICT could be out of scope, reflecting the fact that some family members were generally uninterested in ICT and had difficulties envisioning how it could be used for support. It was also discussed as more of a solution for the future.

**Conclusions:**

Family members described multiple uses for ICT and agreed that ICT could provide access to relevant sources of information from which family members could potentially exchange support. ICT was also considered to have its limitations and was out of scope for some but with expected use in the future. Even though some family members seemed hesitant about ICT solutions in general, this might not mean they are unreceptive to suggestions about their usage in, for example, health care. Thus, a variety of factors should be considered to facilitate future implementations of ICT tools in clinical practice.

## Introduction

### Heart Failure

Heart failure (HF) is a syndrome affecting approximately 1% to 2% of the adult population in most countries of the world. The prevalence increases with age, rising to over 10% in those older than 75 years [1]. Worldwide, HF not only causes considerable suffering for the persons with the condition but also takes up considerable societal economic resources [2]. Having HF affects health through physical symptoms such as breathlessness, fatigue, and swollen extremities [1] and is also associated with psychosocial distress [3,4], for example, depression and anxiety.

### Informal Carers to Persons With Heart Failure

HF deteriorates over time and is unpredictable in its course [1]. HF also impinges on family, friends, and significant others [5,6], especially when the ill person experiences more severe symptoms [7]. Family members often shoulder the role of informal carers, supporting persons with HF both practically and emotionally [5,6,8] and are instrumental in supporting self-care [9] as well as in helping the ill person navigate the health care system [6,9,10].

Informal caregiving can be both rewarding [11,12] and straining [12-14]. Positive experiences of caregiving might include the following: an increase in self-esteem, a feeling of pride [11], and an intensification of the relationship with the loved one suffering from HF [11,14]. Possible negative effects of caregiving are multifaceted [5], ranging from physical, emotional, and social burdens [11] to isolation [5,15]. Informal carers have described feelings of always having to be *on stand-by* [6,10,14] as well as struggling to balance taking care of the ill person, themselves, and maintaining their family and working life [6,10]. It has been established that the needs of carers are not always accommodated [6,16]. In terms of what caregivers do, they describe a need for information and support from health care that is lacking [5,6]. Being a family member or an informal carer of a person with HF could be challenging because of the illness trajectory, where shifts in day-to-day health [6], together with symptoms that will undoubtedly worsen over time, create an unpredictable life situation. This may apply both for those suffering from the syndrome and for family, friends, and significant others. Family members who care for persons with HF are also vulnerable in that they are often older and have health issues of their own [16].

### Information and Communications Technology to Support Informal Carers

Information and communications technology (ICT) could facilitate caregiving, support carers in managing their life situation [17], reduce carers’ burden [18,19], and possibly strengthen self-efficacy [19]. According to a recent scoping review aimed at evaluating Web interventions for informal carers of older persons, carers found ICT feasible and usable. One conclusion was that it is important to adapt interventions to caregivers’ different and changeable needs. However, none of the interventions focused on HF [19]. Providing relevant support for those close to the ill person has the potential to bring positive effects for the family member acting as informal carer as well as the person with HF; however, the use of ICT is relatively unexplored within HF [6].

### Objectives

Knowledge already exists about what needs caregivers might have in relation to the carer role/situation, but there is an apprehended knowledge gap with regard to how family members of persons with HF value and perceive ICT in their everyday life in relation to these needs. The insight into how ICT is perceived by family members could contribute important knowledge that could be used in developing interventions to support informal carers as well as supporting the implementation of ICT clinically. Therefore, the aim of this study was to explore the perceptions of ICT solutions as supportive aids among family members of persons with HF.

## Methods

### Study Design

This study had a qualitative design. Focus group discussions with family members of persons afflicted with HF were performed, and data were analyzed using qualitative content analysis as described by Elo and Kyngäs [20]. The study complies with the Declaration of Helsinki and was approved by the regional ethical review board in Linköping (ref #2015/55-32).

### Participants and Sampling

A purposeful sampling strategy [21] was used with the intention of finding family members from diverse backgrounds, for example, diversity in gender, cultural background, age, and relationship to the person with HF. Initially, patients from an outpatient nurse-led HF clinic at 1 university hospital in Sweden were approached when visiting the clinic, or otherwise contacted by a research nurse. If the patient agreed, he or she was asked to select a family member involved in their care and self-care. Only family members who were able to participate in a focus group interview could be invited, for example, family members who spoke Swedish and had no severe cognitive or hearing impairment. An information letter was sent out or handed to the family member, followed by telephone contact. If the family member agreed to join, a written consent form was signed.

### Data Collection

Before initiating the focus group discussion, participants answered a self-reported questionnaire, developed for this study, about their demographic and clinical characteristics and use of the internet. Participants were informed about the aim and goals of the focus groups. The setup of the focus group discussions was guided by Krueger and Casey [22], and the interview guide had opening, introductory, transition, key, and ending questions to build a logical flow, while also focusing the discussions on support and technology (see Multimedia Appendix 1). Opening and introductory questions were about creating a safe environment. In transition and key questions, participants were asked to reflect on support and to discuss their own experiences of supporting practically, emotionally, informationally, and through confirmation. They were also asked to describe how they cared for themselves in their everyday life. The final key question was a direct question concerning ICT: *What do you imagine internet technology could support you with in your daily life as a family member of someone with HF?* Probing questions, for example, *could you provide concrete examples, could you expand on that, what could help you to provide this care,* were asked to deepen discussions. A pilot focus group was performed to test the procedure, and as no significant changes were made, the pilot group was later included in the analysis.

A total of 2 researchers attended each group and tape-recorded every session, which took place in a secluded meeting room at the university. All the researchers involved in the focus groups (HA, ML, SÅ, and AS) are registered nurses, and 3 out of 4 (ML, SÅ, and AS) have joint positions combining research and clinical work. A moderator (HA or ML) introduced the subjects and questions to be discussed and ensured that all the participants were given a chance to speak. The observer (SÅ or AS) took notes about the discussion and interactions and was able to ask additional questions. The observer was also responsible for producing a summary at the end that the participants could accept, correct, or expand.

A Microsoft PowerPoint presentation with the open-ended interview questions and examples of, for example, existing internet technology solutions were projected during the discussions. These examples included Web meetings, apps, seeking information via the web, and chatting with others. The presentation also included a definition of what a digital service could be about (accessing information without being bound to a certain place and including pictures, films, and text). Participants were reminded to reflect on their role as family members of someone with HF, as opposed to just reflecting more generally on questions. The focus group discussions were followed by a debrief between the moderator and observer to compare understanding of, for example, interactions among participants. All the focus groups were performed between March 2015 and January 2017.

### Analysis

Interviews were transcribed verbatim. To find relevant content and become immersed in the material, the tape-recordings were listened to, and transcriptions read, several times. Afterward, the *unit of analysis* was identified by 2 of the authors (HA and ML). The unit of analysis meant text concerning ICT. The first author then read the unit of analysis several times and started the open coding, making notes and headings in the margins, with the codes then sorted in a coding sheet. The codes and notes were then used to freely generate categories, which in turn were sorted and merged into fewer, more overarching categories. The first author was responsible for developing codes and categories, and another (ML) author verified and suggested changes. This was an iterative and nonlinear process, moving back and forth between part and whole, until no new codes or categories merged. During the process, the authors were careful to stay close to what the participants actually said, while also keeping in mind that an abstraction occurred through the different phases of the analysis. When categories were defined, these and the coding scheme were reviewed by AS and IT for agreement or discussion until a consensus was reached (Table 1).

**Table 1:** Analytical process of focus group data.

Excerpt from unit of analysis	Open coding	Subcategory	Category
“...I think it would be great, it [interacting with other relatives via ICT^a^] would often make it easier. [But] then you realize that everyone is different and a response given to someone may not apply to my husband, but I think that a lot of this with heart failure is...about calming down a bit...”	Even if everyone is different, it might calm worry, to hear other people’s stories.	For exchange with peers and external family	ICT—Providing possibilities for relevant support
“[Discussing having contact with health care personnel via the internet]...those who respond to this could be anywhere, as long as they have the knowledge [Other participant: yes, that's right], so you can get a quick response to see what the possible next step may be [Other participant: mm]...”	ICT is not tied to a specific location; ICT used to write questions to health care professionals and get quick answers.	Unbound by time and place, with endless possibilities	ICT—Providing access
“...so I´d rather meet face-to-face, I want to see that person. I want to see their responses when I ask questions. I think one interprets facial expressions [several other participants confirm] too...”	Wants to see the person when talking to them to be able to read body language.	Physical meetings are irreplaceable	ICT—Being too impersonal
“...it’s too new...we haven’t experienced enough really...”	It is too new [the HF^b^ diagnosis] to be able to see the need for ICT.	Difficult to visualize	ICT—Being out of scope

^a^ICT: information and communications technology.

^b^HF: heart failure.

## Results

### Characteristics of Participants

Participants had diverse backgrounds in terms of, for example, education and occupational status, whereas being more homogenous in terms of, for example, living status and relationship to the person with HF (Table 2). Almost all participants reported having access to the internet, and many used the Web daily. Only 4 had sought information about being a family member or an informal carer (Table 3). A total of 8 focus groups involving 23 family members of persons with HF were conducted. The recordings lasted 55 to 121 min (mean, 91 min). Analysis resulted in 4 categories and 9 subcategories (Table 4).

**Table 2:** Self-reported characteristics of participants (N=23).

Characteristics		Value
Age (years), median (range)^a^		63 (26-85)
**Gender, n**		
	Women	18
**Education, n**		
	Compulsory school	3
	Upper secondary school	8
	University	8
	Other	4
**Main occupation, n**		
	Employed	8
	Self-employed	1
	Student	1
	Retired	12
	On sick-leave	1
Living with a partner, n		23
Living with children, n		6
**Health problems, n^a^**		
	Diabetes, yes	1
	High blood pressure, yes	7
	Rheumatic disease, yes	1
	Stroke, yes	1
	Lung disease, yes	1
	Atrial fibrillation, yes	1
	Myocardial infarction, yes	1
	Other disease, yes	2
**Relationship to person with heart failure, n**		
	Married/partner	22
	Child	1

^a^Some missing values.

**Table 3:** Self-reported internet use among participants (N=23).

Internet use		Frequency, n
Access to internet at home, yes^a^		21
Access internet away from home, yes^a^		15
**How often has the internet been used in the last 3 months^a^**		
	Almost every day	17
	At least once a week	3
	Less than once a week	1
	Do not use	1
**What was the internet used for in the last 3 months**		
	Access to internet banking^a^	20
	Email^a^	19
	Seeking information^a^	18
	Access to news sites^a^	17
	Travel services^a^	16
	Seeking illness-related information^a^	15
	Using apps, music, films, playing games	14
	Selling/buying goods or services^a^	13
	Social media^a^	12
	Video call^a^	10
	Uploading self-made material	8
	Listening to internet radio	7
	Booking appointments for health services	5
	Seeking information about informal caregiving^a^	4
	Creating a website or blog	4
	Playing online games with others	3

^a^One missing value.

**Table 4:** Categories and subcategories from analysis of focus group discussions with family members of persons with heart failure, along with the number of focus groups in which the subcategory occurred.

Category and subcategory		Focus groups, n
**Providing possibilities for relevant support**		
	Interaction with health care personnel	7
	Information and confirmation retrieval	6
	Exchange with peers and external family	7
**Providing access**		
	Unbound by time and place, with endless possibilities	7
	Arena for family members	5
**Being too impersonal**		
	Apprehensions about usability	7
	Physical meetings are irreplaceable	5
**Being out of scope**		
	Difficult to visualize	5
	Something for the future	5

### Information and Communications Technology: Providing Possibilities for Relevant Support

Groups discussed several uses of ICT, including the possibility of communicating with professionals, retrieving information—either to learn about HF/caregiving or to get confirmation of their own thoughts or actions relevant to them. The opportunity to interact with persons in similar life situations, to find and share support, was another subject raised in discussions.

#### Interaction With Health Care Personnel

Almost all the groups discussed the possibility of ICT providing a forum for interacting with health care personnel and receiving individual support. Different aspects of interaction via ICT came up, and being able to ask questions and to receive specific advice was mentioned. Other aspects conveyed were the need for those giving advice or answering questions to have knowledge about HF specifically:

Participant: [discussing communicating with health care] ...If there was a nurse or a physician or a psychologist...who you could ask regular questions and get answers from. I would really appreciate that...

Moderator: ...someone who has specific knowledge...

Participant: Yes...

Being able to put questions to health care personnel specializing in HF was discussed as a way of avoiding information that you do not want or feel the need for. Another aspect raised was the importance of straightforward information that did not hold back on details for fear of causing family members or patients worry.

Web meetings led by professional staff and social media forums were possible arenas for the interaction. It was pointed out that it was important to maintain the privacy of users´ details when arranging closed group sessions on the internet. The importance of Web security was raised in some groups. This entailed both the importance of finding valid sources of information and using secure solutions for communication with health care personnel or other family members through Web-based solutions.

A couple of groups discussed the use of ICT for conveying contacts that could be important to the family member, and it was pointed out that these contacts should be relevant in relation to having HF in the family. One participant put it like this:

We sometimes experience frustration about not knowing where to turn with perhaps quite a simple question.

Another aspect of the interaction with health care personnel was the possibility of using ICT for follow-up of the person with HF. This could potentially unburden the family member. Other positive examples of helpful solutions were remote rhythm monitoring of the person with HF living with an implanted cardiac defibrillator, and the possibility of following up on hospital visits to deal with questions arising after a visit to the physician, for example.

#### Information and Confirmation Retrieval

According to the family members, ICT offered a way to retrieve information about HF-specific matters, and several groups discussed already having used this possibility. Several practical examples of HF-specific information for family members were given. The content presented through ICT could entail information about symptoms of HF, the consequences of symptoms, and warning signs—what to look out for in the ill person: (information about)...*warning signs* (relating to HF) *that you should look out for, that could be of help.* Information concerning care trajectories was also mentioned as well as information on heredity. Another topic concerned cardiac-related anxiety and how to separate this from actual symptoms of HF, and how to tackle this as a family member. ICT could also be used to convey concrete information and support concerning what family members might encounter when the person with HF is in hospital. A wish to read about HF in younger persons—about how it is possible to have a good life even though illness is present in the family—was also conveyed. Yet another aspect was that it would be supportive, if advice about what to eat, were translated into concrete menus. Family members also wished to receive discharge notes aimed specifically at them. ICT could also involve suggestions about questions to ask during visits to health care institutions.

The possibility of ICT being used to organize information in a comprehensible and easy-access format was also mentioned. It was suggested that the need for information was related to the symptoms of the ill person. When the family member was first diagnosed, it was important to read about medicines and side effects, but when the person with HF was in a stable phase, this need did not seem equally apparent.

ICT could also be used to receive confirmation and support decision making—information through ICT could help determine whether to contact health care or not:

...when something happens at home then you go to the internet and look [other participant hums]...before deciding to call 1177 [a national telenursing service number] [laughs] you could do a bit of research on your own...

Confirmation of the family member´s own actions and support in relation to the ill person could help to reassure the family member.

Some spontaneously discussed how information and confirmation could be delivered through text on a Web page, through a recorded lecture, a frequently asked questions section, and through social media forums. It was also stressed that information on the Web should be presented so that everyone could understand, meaning that not too many medical terms should be used.

#### Exchange With Peers and External Family

ICT could be useful for contact with other family members of persons with HF. This seemed to have social, practical, and emotional functions, and interacting with other family members could entail a learning experience as expressed by this participant:

...you can read the posts [on the internet] and maybe learn something from it. Because it’s a bit difficult and you will never have learnt enough as a next of kin...

Contact with other family members could also calm worry and support problem solving. Receiving advice from others, for example, on what to consider in specific situations, as well as the possibility of sharing one’s own experiences, was discussed. Connecting with other family members through ICT seemed to be a way of recognizing oneself in others.

The opportunity to connect with others in similar life situations could also be valuable for the persons with HF, and it could be important to have separate groups for family members and persons with HF as well as mixed groups:

[Answering a question from the moderator about who a social media group would be aimed at, the patient or the family member?]...I think, both, but at the same time you also need to be able to talk about them [referring to their family member with HF], even though they don’t always know...so both...because they also need to talk about it [referring to person with HF].

Also mentioned was the fact that it could be easier to *talk via the Web* if you had met the person in real life first, or that connecting with others through ICT could be a prerequisite for seeing each other in real life later.

Chat groups and social media forums were raised as possible Web-based solutions for interaction with other family members.

A few groups also discussed how ICT could be used for reaching or keeping in contact with extended family. The use of social media could, for example, mean that one did not have to have personal contact with everyone in the family in times of crisis. By posting information on social media, you could relieve yourself of having to answer questions from extended family, thus enabling you to focus on the ill person. On the other hand, the possibility of maintaining personal contact was also described as a strength of ICT.

### Information and Communications Technology: Providing Access

ICT could offer solutions accessible from anywhere and whenever, and ICT could be both timely and adaptable to different life situations. ICT could also provide an arena for family members that they might not have had access to otherwise.

#### Unbound by Time and Place, With Endless Possibilities

Groups discussed how ICT allowed the option of adjusting times when information could be accessed and provided via the Web. This was mentioned as a pro for, for example, family members who worked and for those with little spare time during regular working hours.

The groups discussed how ICT was unbound to a specific place when accessing or providing information, something that was also mentioned as being favorable for health care personnel. Remote solutions, for example, checking blood pressure, weight, and symptoms and having an initial assessment done via an internet-based communication tool were mentioned as one possibility. ICT was also stated as a solution that would bring health care closer to the patient and family member:

I think that taking the doctor or the care to him [the patient, through ICT] would be very useful and then I could feel that I can relax a little...

It was also suggested that ICT could provide rapid answers to questions and concerns and be something that could give access to *everything*. When discussing this, it was not always explicitly expressed *how* ICT had a specific use for the family members. These statements seemed to be more about showing an understanding that “you have the world in your pocket” when having access to the internet, and if you know where to look and have the knowledge to use computers, you have access to everything:

...if you have an internet connection, you actually have all the possibilities in the world...[another participant confirms].

#### Arena for Family Members

ICT could provide an arena for the family member to take part of information that concerns the person with HF but also for addressing one’s own questions. Family members did not always feel they were able to take up space in health care consultations:

It concerns him [referring to the ill person]...so you don’t have that space as family at all...

Some discussed opportunities to ask questions without their ill family member present; however, others thought it was important that both the person with HF and the family member had access to the same information.

ICT could increase their participation in caring for the ill person. Receiving information about the person with HF while he or she was hospitalized—even when not being able to be present—could ease involvement:

...I would like to be...a little bit more involved. Because I was not physically here [at the hospital] at these times...one could have received documentation [concerning status]...[other participants confirm]. It would have been very easy, either by e-mail or by regular mail.

### Information and Communications Technology: Being Too Impersonal

ICT was perceived as entailing limited personal interaction and individualization, which could cause mistrust of information and could cause a feeling of ICT being less usable. It was made clear in several discussions that ICT could not replace physical meetings.

#### Apprehensions About Usability

It could be hard to recognize oneself in information provided through ICT, as expressed by this participant:

...it rarely fits you directly [discussing Web-based information], or maybe you imagine that it does, but it doesn´t really...

Groups discussed that ICT could not be used for everything, for example, for support in acute situations or for dealing with worry:

...well, a disadvantage of the chat function is that I can’t convey the worried feeling I might have...

Concerning acute situations, an app for support in the event of a cardiac arrest was mentioned. Even though this app was recognized as a valid alternative, it was also implied that it was not necessarily trusted.

Some discussed how information on the internet could provide answers to *some* questions but that it was seen as being too based on facts and not personal enough for everything. This indicated a need for personalized and tailored information and was something that could get in the way of ICT being a usable aid for support.

Other aspects were that ICT was not personalized enough. Some family members also discussed how seeking information through ICT could cause worry and lead to imagining illnesses or problems that were not there:

...I think that you eventually only get sicker and sicker if you read everything, and more worried too.

Even though groups discussed the supportive effect of communicating with other family members through ICT, some were hesitant about the idea of connecting with other family members via the Web. Some questioned meeting *strangers* via the Web, and others discussed how communicating through ICT could mean that discussions might *derail* and that some persons could *show too much of themselves.* In connection to this, it was mentioned that contact with other family members via ICT needed to be organized to avoid this.

#### Physical Meetings Are Irreplaceable

ICT could not replace physical meetings. The reasons for this were already having the necessary contacts with health care personnel in real life or not feeling a need for ICT. Some just stated that they prefer to see someone “eye to eye” instead of receiving information via ICT:

I don’t want information via the internet, I want real information.

Not allowing body language to be read was given as a disadvantage of ICT. Another reason for preferring physical meetings was that ICT as an aid would not be able to relieve worry.

### Information and Communications Technology: Being Out of Scope

ICT was described as being out of scope, and some were generally uninterested or had less faith in ICT. They did not know much about ICT and were less interested in using it. For some, it seemed hard to envision what ICT could be of use for, in relation to being a family member of someone with HF, and it was thought of as more of a solution for the future.

#### Difficult to Visualize

Groups expressed a difficulty in envisioning how ICT could support them in their everyday life. This became clear when groups were approached with questions concerning ICT during the focus group discussion but stated that they did not think they could contribute:

I can’t think of anything right now, because my first thought was that the internet...couldn’t help me with anything...

It could be that being a family member of someone newly diagnosed with HF also made visualization difficult. However, when presented with examples of what ICT could be about during the focus group discussions, it seemed that some family members took inspiration and incorporated these into their discussions.

#### Something for the Future

ICT was considered in some groups to be something suited more for the future and for coming generations but also as something that could be useful if the ill person’s health deteriorated. Groups discussed how coming generations might be better equipped to use these kinds of solutions, and it was mentioned that younger generations already had ICT as a natural part of their daily life. Some groups discussed the significance of age and how it could be that they were too old for ICT:

One can say that I belong to the older generation, and it is more our children and grandchildren who will use it [referring to using a computer] and get more benefit from it I would think...so in the future...

Not everyone agreed, and in response to one family member expressing being too old for ICT, another participant responded by pointing out that:

You’re never “too old to learn something new.”

## Discussion

### Principal Findings

The analysis explored perceptions of ICT solutions as supportive aids among family members of persons with HF. Overall, ICT was seen as having a broad range of uses, while also involving—or lacking—content or qualities that might make ICT less useful for family members of persons with HF.

### Comparison With Prior Work

Participants suggested that ICT could be used for interacting with health care personnel, to give and receive support from other family members, and for communication with the extended family. The interaction with health care personnel was considered to be useful for receiving personal advice from knowledgeable experts, whereas interaction with other family members seemed to be about recognizing oneself in others—and receiving emotional support and advice. The importance of being able to communicate with others in similar situation [23] and health care personnel [24] is reflected in other studies, and connecting with other carers via the Web has been noted as being just as helpful as seeing someone physically [19]. Although our study suggests that for some carers, ICT could not replace physical meetings and could even cause worry, communication with both health care professionals and peers via the Web seemed to be of interest.

In developing support through ICT, there could be a challenge in balancing relevant information with specific information that will not evoke negative feelings. Some participants stated that seeking information on the internet could have negative effects, such as causing anxiety and worry. For some, it was considered important to have a personal contact with the health care personnel to obtain individualized information, whereas others seemed to want both personalized and general information. The need for both types of information mirrors other findings [25]. The need for different kinds of information might also relate to different coping styles, with coping strategies such as being a *monitor* or being *a blunter* impacting on what is considered relevant. Being a monitor or a blunter in relation to use of information has been studied previously [26-28]. It could be that a person who is mainly characterized as a *blunter* avoids threatening information [26] and, therefore, might not seek information via the Web. For those labeled a *monitor* (wanting and scanning for information while also being more anxious) [26], information delivered through ICT could cause added stress if not carefully worded. Factors such as these could be of importance when producing Web based informational material. Questions such as—whom will it actually reach and how could the information affect those reading it, needs to be addressed. When producing material, it may also be worth considering adapting information to the relationship that the family member has to the ill person [29]. In this study, we almost exclusively had discussions with spouses and it might be that, for example, siblings or friends may suggest other uses of ICT.

In our study, some family members felt that ICT could not replace physical meetings, for example, because it hindered the reading of body language and also as it could be difficult to convey worry. This might not necessarily reflect a limitation concerning ICT but rather the fact that it was hard for some family members to visualize how ICT could be a support. In a study concerning ICT support for carers, the conclusion was that when the elderly carers received support from the nurses, they were more likely to use the internet-based intervention [15]. It might be that the initial reaction to suggestions about using ICT for support is not necessarily unsusceptible to influence, and it could therefore be important to educate and support carers in how to operate ICT when implementing these kinds of interventions.

Almost all the participants in the focus groups had access to and used the internet on a daily basis. Many also reported having access away from home (eg, via a mobile phone). Even so, not all expressed an interest in the use of technology in their everyday life. Some said they were too old for ICT, and some expressed a lack of interest or even mistrust of technology, or that they had enough of computers and technology from work. In a previous study, investigating women who chose *not* to participate in screening for colorectal cancer, data indicated that only 1 in 4 actually made use of information presented on the Web. When looking for reasons for this *nonuse*, the only association found was between age and actual Web use. This meant that in older age groups, it was less likely that the women had accessed the information delivered through ICT [30]. The same result, concerning age and ICT, was also indicated in other studies on family members of persons with cancer [31,32]. This might partly reflect the fact that the elderly of today have less experience of using technology than future groups will have and that it is this not only age per se that is hindering the use of ICT. A recent study investigated what might affect the adoption of different technologies (not only ICT) in the elderly, and a factor that seemed important was the *perceived value* [33]. A reflection in relation to this is that, what may be seen as valuable, might also reflect general experience and expectations concerning ICT, and this in turn could be affected by age in today’s elderly. Introducing ICT as an intervention aimed at family members might be complex, and *access* may not be the only thing to consider—especially in countries such as Sweden where 94% to 100% of persons aged 16 to 64 years have access to the internet in their homes. This number decreases with older age groups, but still, 86% of persons aged between 65 to 74 years and 68% of persons aged between 75 to 85 years report having internet access in their homes [34].

We found that some family members seemed to be influenced by examples of ICT use, and it might be that, when introduced to ICT, some elderly persons or those with the traits of a *blunter* might need extra support in seeing the usefulness of it and also actually using it. Factors such as age, coping style, perceived value, interest in, and knowledge about ICT could be of importance when developing, introducing, or implementing ICT-based support.

### Limitations

This study was performed in 1 center in 1 country, which may limit transferability to other settings and cultures. However, we believe that some of the experiences may be universal and also apply to caregivers of patients with other chronic conditions. There was a variation in group size between the 8 focus groups. A total of 6 groups had 2 participants each, 1 group 3, and another 8. In the literature [35], it has been discussed that it is the involvement from participants that matters more than the actual group size. Focus group studies entailing only 2 members have been published before [36], and considering the aim and data, we found that group sizes gave relevant data. No new subcategories emerged in the last focus group, which indicated that we had sufficient material to mirror Swedish-born, married/cohabitant caregiver’s perceptions of technology from the 8 group discussions.

All researchers, except the main author, were experienced in working with focus group discussions, and there were no significant relationships between researchers and participants.

All but 1 participant was a spouse or partner to the person with HF, and all the participants were of a Swedish background. Most participants were women, and this reflects the population of older family caregivers in particular. The groups reflect different educational background and employment status. It was not possible to recruit participants according to the initial plan, and it might be that carers with other relationships to the ill person and with a more diverse background could have nuanced the results even more. Even so, heterogeneity and homogeneity concerning composition of focus groups has been problematized, and it seems that *commonality* in focus groups is important for capturing the shared experiences and that having too much spread in groups could hinder discussions [37]. We therefore consider the partially homogeneous groups as both a weakness and a strength.

Family members in this study did not always see themselves as caregivers but rather as family. It is possible that family members who lived/supported someone with a more severe HF could more easily have expressed needs in relation to ICT. At the same time, the person with HF defined who to invite to participate in the focus groups, and this strengthens the possibility that our data reflect the general family member population.

### Conclusions

Family members described multiple uses for ICT, and agreed that ICT could provide access to relevant sources of information from which family members potentially could exchange support. ICT was also considered to have its limitations and was out of scope for some but with expected use in future. Even though some family members seemed hesitant about ICT solutions in general, this might not mean they are unreceptive to suggestions about their use in, for example, health care. Thus, a variety of factors should be considered to facilitate future implementations of ICT tools in clinical practice.

## Appendix

Multimedia Appendix 1 Semistructured interview guide describing opening, introductory, transition, and key questions.

## Abbreviations

HF: heart failure
ICT: information and communications technology
