# Supplementary material for: Perceptions of Information and Communication Technology as Support for Family Members of Persons With Heart Failure: Qualitative Study
Source: J Med Internet Res. 2019 Jul 16;21(7):e13521. doi: 10.2196/13521 (PMC6664659; doi:10.2196/13521)
Supplement: Multimedia Appendix 1 [file jmir_v21i7e13521_app1.pdf]

Interview guide

- Tell us your name and also something about your relationship to the person with heart failure close to you…
- Can you tell us about your everyday life as a family member of someone with heart failure?
- What do you think supporting someone might involve?
- Do you have any experience in providing practical support for your family member?
- Do you have any experience in providing information to support your family member?
- Do you have any experience in strengthening or confirming your family member in what is said or done?
- Is giving emotional support something that you have experience of?
- Has your role as an informal carer changed over time?
- What do you do in everyday life to stay healthy/take care of yourself?
- What do you imagine internet technology could support you with in your daily life as a family member of someone with heart failure?
